# Synergistic Optimization of Plasma-Sprayed Zirconia Coatings: Towards Ultra-High Hardness and Superior Wear Resistance

**DOI:** 10.3390/ma19163478

**Published:** 2026-08-17

**Authors:** Shengyu Chen, Mengya Chen, Qiduan Chen, Mingder Jean, Weimin Luo

**Affiliations:** 1 College of Arts and Design, Jimei University, 185 Yinjiang Rd., Jimei District, Xiamen 361021, China; 202221274045@jmu.edu.cn (S.C.); qdchen@jmu.edu.cn (Q.C.); 2 Shanghai Academy of Fine Arts, Shanghai University, 99 Shangda Road, Baoshan District, Shanghai 200444, China; mengya@shu.edu.cn

**Keywords:** zirconia, plasma spray, microhardness, wear volume losses, orthogonal array, response surface method, desirability

## Abstract

This study reported on the multi-objective optimization of atmospheric plasma spraying parameters for zirconia coatings by incorporating the response surface method with the desirability method, while simultaneously enhancing microhardness and reducing wear rates. The spraying variables were optimized to improve coating performance using an L18 Taguchi orthogonal experimental design combined with the response surface method. The analysis of variance revealed the primary factors to be acceleration voltage, stand-off distance, powder feed rate, and primary gas Ar/H_2_; together, these factors accounted for 79.70% of the total performance variance. An R^2^ value of 0.752 for microhardness was yielded by the fitted reduced quadratic model, whereas an R^2^ value of 0.845 for wear volume losses was yielded by the interaction model. Within the optimised parameter range, the predicted microhardness was 1404.1 HV, with an experimental error of only 1.84%, and the wear volume loss was reduced to 4.53 mm^3^, with a prediction error of 3.27%. Additionally, the optimized coating was found to have fully molten droplets, less porosity, and negligible interlayer cracks, with only slight pitting observed during the wear test, as revealed by scanning electron microscopy. This multi-objective optimisation strategy, integrating the Taguchi method with the RSM, effectively enables the preparation of zirconia coatings with superior mechanical properties and provides reliable process guidelines for protective coatings on aluminium alloys.

## 1. Introduction

A variety of processes are employed to produce coatings for specialised applications, such as welding, plasma spraying and surface hardening [1,2,3,4]. These techniques form functional layers on the substrate surface, thereby improving its metallurgical properties (interlayer bonding, interfacial adhesion, residual stress uniformity and phase stability), which further enhances its mechanical and tribological performance including microhardness and wear resistance. Plasma spraying is a particularly versatile process for producing protective coatings and has therefore attracted widespread attention [5,6,7,8,9,10]. The process has many benefits, including being low-cost, having moderate operating temperatures, being easy to prepare, and resulting in high coating quality [11,12,13,14,15]. Furthermore, the required wear resistance is achieved while less coating material is used. Because of these benefits, there is a lot of research into plasma spraying coatings because they are a cost-effective solution in numerous industrial applications [16,17,18,19,20].

Although manufacturers have developed and industrially applied plasma-sprayed ceramic coatings since the mid-20th century, researchers have still focused extensively on researching them in recent decade [21,22]. Zirconia-based coatings are widely used for the protection of metal components, where they significantly enhance wear resistance and thermal insulation [23,24]. Zirconia ceramics are predominantly used in the production of hard, wear-resistant coatings, for which surface characteristics are of critical importance. Numerous studies have demonstrated that plasma-sprayed zirconia ceramic coatings exhibit highly promising properties [25,26,27]. These studies have focused on issues such as residual stress, porosity, cracking, deformation, microstructure, thickness, parameter control, bond strength between the coating and the substrate, and delamination [28,29]. Among these studies, the existing literature primarily focuses on plasma-sprayed coatings made from highly wear-resistant materials. Despite the fact that these coatings have the potential to improve the microstructure of the substrate surface and enhance properties such as wear resistance, corrosion resistance, oxidation resistance, and thermal shock resistance [30,31], there are still many challenges that need to be addressed. Moreover, most studies to date have focused solely on the individual properties of zirconia-based coatings produced by plasma spraying, while paying little attention to their multifunctional behaviour. As a result, the overall performance of these coatings remains insufficiently understood, which limits their effective utilisation in industrial applications. As the manufacturing industry becomes increasingly diverse, multiple types of responses often occur simultaneously alongside multi-parameter effects [32,33]. These responses are influenced by numerous factors, making further analysis necessary.

A large number of studies have examined the relationship between plasma spraying process parameters and the mechanical properties of zirconia coatings. Numerical modelling of plasma spraying processes is of paramount importance, particularly in high-speed industrial production [34,35,36]. However, controlling coating quality using this technology is extremely challenging due to the interactions between operational parameters and the numerous uncertainties in the manufacturing environment. Despite the wealth of literature exploring the relationships between process parameters, interactions and coating properties, traditional modelling approaches still struggle to elucidate these relationships. However, most existing studies on plasma-sprayed zirconia coatings rely on trial-and-error methods, which fail to reveal the interactions among multiple process parameters and their combined effects on the surface properties of the coatings [37,38,39,40]. There is a significant challenge in establishing an appropriate model with multiple-response properties because of the inherent characteristics of the spraying process and the variations in multiple parameters in the manufacture of plasma-sprayed ceramic coatings [41,42,43]. Nevertheless, there are alternative strategies that are expected to address the issues associated with the multiple responses of plasma-sprayed deposits [44,45,46,47,48].

The challenges of process optimisation involving coupled multi-parameters with multiple responses are addressed by this study. It introduces methods such as the Taguchi method and response surface methodology to the field of plasma spraying coatings in surface engineering [49,50,51]. The Taguchi method uses orthogonal arrays to significantly reduce the number of experiments required for identifying key influencing factors, while the RSM constructs continuous mathematical models between multiple variables and response outputs to allow for the precise determination of process parameters that yield optimal responses. By using the methods described above, factors that are important can be identified quickly, and models that can predict coating properties and mechanical performance with a high precision can be developed. This provides an efficient and feasible way of improving the performance of plasma-sprayed coatings.

This study aims to enhance solutions for multiple responses such as maxima hardness and minima wear volume losses by RSM with the desirability method, based on Taguchi design. Meanwhile, the spraying process for zirconia coatings, which exhibit multiple response characteristics, has been optimized, and the influence of spraying parameters on coating properties has been investigated, thereby contributing to the improved mechanical properties of the coatings. In this study, the mechanical properties examined were limited to hardness and wear rate. To address the issue of these multiple quality attributes, the desirability method of RSM was employed. Two-dimensional response surface plots are generated to visualize the individual and interactive effects of the parameters on the responses. Furthermore, multi-objective optimization was carried out using the desirability-overlapped function approach to identify the optimal parameter window that satisfies both high hardness and low wear volume losses. To overcome the limitation of existing studies optimising only a single coating property, this work presents a synergistic, multi-response optimisation strategy. The predictive capability of the regression model is verified through systematic microstructural and tribological characterisations. The model can reliably predict how well plasma-sprayed ceramic coatings will perform. These coatings are resistant to wear and tear. The aim is to enhance production efficiency during the transition to large-scale industrial production.

## 2. Experiments

### Materials and Preparations

In this study, the ceramic coating was prepared via plasma spraying using Y_2_O_3_-stabilized zirconia (YSZ), a commonly used thermal spray coating material. Such coatings are widely used in thermal insulation and protective applications. The commercially available sintered, agglomerated yttria-stabilized zirconia powder supplied by Sulzer Metco (Winterthur, Switzerland) was adopted as the coating feedstock, with an overall particle size of 80–120 μm, as well as particle size distribution parameters of d_10_ = 32 µm, d_50_ = 68 µm, and d_90_ = 105 µm. The powder exhibits typical spherical agglomerated morphology formed by micro-scale primary zirconia crystallites. It contained 90.28% ZrO_2_, 7.52% Y_2_O_3_ and trace amounts of other components (Al_2_O_3_, SiO_2_, TiO_2_, Fe_2_O_3_, CaO and MgO). The equipment used for spraying uses a plasma spraying system with an F4 plasma spray gun and a powder feeder, which is also made by Sulzer Metco. The raw material was dried at 120 °C for two hours before spraying to remove moisture. Aluminium alloy 6066 was selected as the substrate material due to its widespread industrial use [26]. It offers high strength, excellent corrosion resistance and superior mechanical properties, making it ideal for high-stress applications. The substrate material was aluminum-based alloy, machined into 150 mm × 100 mm × 10 mm specimens. The substrate surface was sandblasted with 60-mesh Al_2_O_3_ particles pre-spraying to achieve an arithmetic mean roughness of approximately 4.5 μm. It was then ultrasonically cleaned in ethanol for 15 min to remove surface contaminants. Only one coated specimen was manufactured for each orthogonal parameter combination. Different areas of the same sample were tested for microhardness and wear several times, with no replicated coatings prepared. The microhardness of the surface was measured. This was done using a micro-Vickers hardness tester (HVS-1000Z, Huazheng Electric, Baoding, China). A load of 2.94N was applied for 10 s. A total of seven indentations were tested on each coating cross-section and the average value of these was calculated to determine the final hardness. The structure of the spray-coated Y_2_O-stabilized ZrO_2_ coating was analysed using a Hitachi S-2600H scanning electron microscope (Tokyo, Japan) and an energy-dispersive X-ray spectrometer. In addition, the phase composition of the deposits was measured using a Philips X-ray diffractometer with copper (Cu) Ka radiation across a 2θ range of 20–80°. Coating thickness was determined by SEM on polished cross-section specimens. To reduce local-region bias, thickness measurements were taken from six random positions across the coating cross-section. In addition, a series of wear tests were conducted on the ZrO_2_ coating. All samples in this study were ground before the tests. The tests included a 1000 m test using a ball-on-disk wear tester and a WC ball as the counterpart. During the wear test, the counterpart ball moves around the center of the disk. The disk remained stationary while the WC ball moved along a circular path on its surface. The parameters of wear test were as follows: a test load of 10 N, a sliding speed of 30 cm/s, wear track diameters of 20 mm and a sliding distance of 1000 m. The depth of the wear tracks was measured and subsequently the wear volume loss was calculated using a TalyScan 150 (Taylor Hobson, Leicester, UK) surface profilometer. An L18 mixed orthogonal array was used to identify eight plasma spraying process control factors. Key process parameters were examined in the research, including coating layers (6–8), accelerating voltage (65–75 V), arc current (A), travel speed (300–500 mm/s), stand-off distance (8–12 cm), powder feed rate (20–30 g/min), carrier gas flow rate (Ar) (20–30 L/min), and primary gas Ar/H_2_ (50–60 L/min),. Factor A had two levels, while factors B–H all had three. This design required only 18 runs to explore the main effects of the various parameters and some of their interactions, which significantly reduced the cost of the experiment. In addition, the performance of the coating is evaluated based on its surface hardness and volumetric wear loss properties.

## 3. Experimental Design and Analysis

### 3.1. Control Factors and Their Levels

The parameters for this study were determined based on expert experience and literature reports [3,4,5]. In the experiments, eight controllable operational parameters were listed on the left side of the orthogonal array, including the number of coating layers (A), accelerating voltage (B), arc current (C), travel speed (D), stand-off distance (E), powder feed rate (F), carrier gas flow rate (G), and primary gas Ar/H_2_ (H); these parameters are detailed in Table 1. The implementation of orthogonal array-based Taguchi methods has the potential to curtail the time and expense demanded by experimentation. These arrays provide a balanced experimental design covering a large number of design factors, while fulfilling the signal-to-noise ratio (SNR) requirements of Taguchi methods [10]. The experiment employed an L18 orthogonal design. The levels for each parameter are shown in the lower left corner of orthogonal array as shown in Table 1. Additionally, the experimental results for the plasma-sprayed wear-resistant ZrO_2_ coating, including microhardness (HV) and wear volume losses (mm^3^), are listed on the right side of Table 1. Both the microhardness tests, repeated seven times, and the wear volume tests, repeated three times, are shown with their respective mean values and standard deviations. The calculation of the Taguchi SNR is displayed on the right-hand side of the table. The SNR for how hard the coating is and how much it wears is worked out based on the idea that bigger is better and smaller is better. The utilisation of SNR for the purpose of evaluating quality results in an emphasis on variability. Additionally, it assists with data analysis, parameter optimisation and result prediction.

### 3.2. Response Surface Methodology

Response Surface Methodology (RSM) is a set of statistical and mathematical techniques used to model and optimize target responses influenced by multiple independent variables [33]. In this study, the RSM was employed to investigate the effects of key spraying parameters on coating microhardness and wear volume loss. The optimal process conditions that can simultaneously improve both of these properties, such as achieving maximum hardness and minimum wear characteristics, were determined. The relationship between the response and the variables can be modelled, which helps us to understand the extent of their influence. In addition, graphical maps of geometry and contour plots can be made available. These are visual illustrations. They help us to better understand the behaviour of the relationships. Model-type attitudes are generally unknown, whereas the empirical model is used in the region of the independent variable of interest. The yield obtained by the process is expressed in terms of the x_1_, x_2_, … and x_n_ parameters, which can be expressed as follows:

(1) $$y\;=\;f(x_{1},\;x_{2},\;\ldots \;x_{n})\;+\;\varepsilon$$

where ɛ denotes the observed error in the response variable y and x_i_ are the independent variables, f is a first- or second-order model. The fitted polynomial model can be written in matrix form as follows:

(2) $$Y\;=\;\beta_{0}\;+{\;X}^{T}b\;+\;X^{T}BX\;+\;\varepsilon$$

where

$$Y=\left[\begin{array}{c} y_{1}\\ y_{2}\\ \vdots \\ y_{n} \end{array}\right],\;b=\left[\begin{array}{c} \beta_{1}\\ \beta_{2}\\ \vdots \\ \beta_{k} \end{array}\right],\;B=\left[\begin{array}{cccc} \beta_{11} & \beta_{12}/2 & \ldots & \beta_{1k}/2\\ \beta_{22} & \ldots & \beta_{2k}/2 & \\ \vdots & \vdots & \ldots & \vdots \\ \mathrm{sym}. & \ldots & \beta_{kk} & \end{array}\right],\;X=\left[\begin{array}{c} x_{1}\\ x_{2}\\ \vdots \\ x_{k} \end{array}\right],\;\varepsilon =\left[\begin{array}{c} \varepsilon_{1}\\ \varepsilon_{2}\\ \vdots \\ \varepsilon_{n} \end{array}\right].$$

In addition, the mean of ***Y*** is:

$$E(Y)\;=\;\beta_{0}\;+{\;X}^{T}b\;+{\;X}^{T}BX.$$

To get the best possible prediction for **Y**, we use the least squares approach to find the regression parameter **β**. The least-squares estimate of the vector ***B***, also known as by $\hat{B}$, makes the predicted values pretty similar to the observed values, and it also minimises the sum of squared errors (SSE), which is expressed like this:

(3) $$\mathrm{SSE}\;=\;\sum_{i=1}^{n}[Y_{i}\;-\;E(Y_{i}){]}^{2}\;=\;[Y\;-\;(\beta_{0}\;+\;X^{T}b\;+\;X^{T}BX){]}^{T}[Y\;-\;(\beta_{0}\;+{\;X}^{T}b\;+\;X^{T}BX)]$$

The application of the techniques in matrix algebra to the minimisation of (3) yields

$$\hat{b}=\left[\begin{array}{c} {\hat{\beta }}_{1}\\ {\hat{\beta }}_{2}\\ \vdots \\ {\hat{\beta }}_{\kappa } \end{array}\right],\hat{B}=\left[\begin{array}{cccc} {\hat{\beta }}_{11} & {\hat{\beta }}_{12}/2 & \ldots & {\hat{\beta }}_{1k}/2\\ {\hat{\beta }}_{22} & \ldots & {\hat{\beta }}_{2k}/2 & \\ \vdots & \vdots & \ldots & \vdots \\ sym. & \ldots & {\hat{\beta }}_{kk} & \end{array}\right]=(X^{⊤}X{)}^{-1}X^{⊤}Y$$

provided that the matrix **X**^T^**X** is non-singular. Consequently, the predicted value of Y_i_, as determined by the fitted regression model, is calculated as follows:

(4) $${\hat{y}}_{i}=\;{\hat{\beta }}_{0}+{\sum_{i=1}^{k}\hat{\beta }}_{i}x_{i}+{\sum_{i=1}^{k}\hat{\beta }}_{ii}x_{i}^{2}+{\sum_{i\leq j}\sum_{i\leq j}\hat{\beta }}_{ij}x_{i}x_{j}.$$

The quadratic equations of the response surface above are established using Taguchi design. This makes the design matrix for experiments more efficient. It is evident that in this particular study, an orthogonal array table from Taguchi’s design was utilised. A mean and variance table was used to select important factors. We applied RSM to compute the coefficients of this response model, for which Design-Expert 8.0.6 software (Stat-Ease Inc., Minneapolis, MN, USA) was used to perform the RSM calculation and various statistical analyses were conducted to validate the model.

## 4. Experimental Results and Discussion

### 4.1. Surface Performance of Coatings

Table 1 lists the L18 orthogonal experimental design, which includes eight plasma spraying parameters, the mean microhardness, the standard deviation, and the SNR for each trial. Microhardness was measured at seven locations on the cross-section of the coating, while the SNR was calculated based on the higher-is-better attribute to simultaneously evaluate both microhardness performance and stability. Based on the results of hardness tests conducted on plasma-sprayed coatings and the calculation of SNR values, the surface microhardness values ranged from 890 ± 160 to 1510 ± 513 HV. This resulted in an average value of 1165 HV and a standard deviation of 209 HV. This wide variation in microhardness demonstrates that process parameters significantly impact microstructural densities. The results of the tests on the plasma-sprayed coating, along with the calculations based on the SNRs, are shown in Table 1. The highest microhardness in the entire table was found in Test 6 at 1510 HV, with a standard deviation of 513 HV, indicating extremely poor uniformity of coating and placing it in the group with abnormally high microhardness. The XRD peaks of the test are found at 30.3° t-ZrO_2_ (101), 34.8° ($\bar{1}$02), 50.4° (122) and 60.1° (302) [51]. This high microhardness may be due to locally dense areas, whereas unmelted zones result in low microhardness, rendering the coating unsuitable for mass industrial production. Test 16 exhibited the second highest microhardness in the table at 1490 HV with a standard deviation of 98 HV, demonstrating high microhardness and excellent uniformity. The XRD peaks of the test are found at 30.3° t-ZrO_2_ (101), 33–35° t-ZrO_2_/c-ZrO, 40–42° t-ZrO_2_, 50–52° (122) t-ZrO_2_/c-ZrO_2_ and 58–62° (302) t-ZrO. Microstructural analysis revealed that the powder had melted completely, the flat particles were evenly distributed, interlayer bonding appeared very strong, porosity was minimal and there were no obvious unmelted particles to be found. In contrast, the microstructure of test 14 shows that the ZrO_2_ powder was not fully melted, and that there were a large number of unmelted particles. There also appeared to be a significant number of pores and microcracks between layers, which caused its microhardness to drop sharply. Additionally, as shown in Table 1, the volume loss of plasma-sprayed ZrO_2_ coatings due to wear varied significantly, ranging from 3.07 mm^3^ to 12.63 mm^3^. This difference was more than fourfold under different parameter conditions. This indicates that the wear resistance of the coating varies significantly depending on the spraying conditions. The lowest volume loss during wear (3.07 mm^3^) was shown by test 11, indicating the best wear resistance. The XRD peaks of the test are found at 30.3° t-ZrO_2_ (101), 33–35° t-ZrO_2_/c-ZrO, 40–42° t-ZrO_2_, 50–52° (122) t-ZrO_2_/c-ZrO_2_ and 58–62° (302) t-ZrO. The material is likely to have a dense coating structure with few pores and no unmelted particles. This, coupled with strong interlayer bonding, leads to a wear mechanism that is dominated by minor abrasive wear. In contrast, test 14 exhibited the highest volume loss (12.63 mm^3^), which corresponds to the poorest wear resistance. It appears to have a structure containing numerous unmelted particles and high porosity, as well as weak interlayer bonding. It was found that the XRD peaks of this test were similar to those of test 11, but only at 30.3°, and that t-ZrO_2_/c-ZrO_2_ is different. This results in coating spalling, particle detachment and abrasive scraping during wear, leading to a sharp increase in volume loss. Based on the microhardness and wear volume data presented above, test11 exhibits moderate microhardness (1151 ± 159HV) but the highest wear resistance (3.07 mm^3^). This indicates that interlayer bonding and structural densities are more important than microhardness alone. Test 6 had the highest microhardness (1510 ± 513 HV) but extremely poor wear resistance (11.19 mm^3^). This may be due to high microhardness combined with high brittleness and numerous defects, making it prone to spalling. By contrast, test 16 exhibited high microhardness (1490 ± 98 HV) and low wear (3.82 mm^3^), highlighting the optimal balance of high microhardness and high wear resistance in the table. It is noteworthy that high microhardness does not always guarantee high wear resistance. In any case, it is only feasible to optimize a single property rather than multiple properties simultaneously. The properties of the aforementioned coating cannot be directly applied to industrial production. Further post-processing is required.

### 4.2. Surface Methodology of Coatings

**Figure 1:**
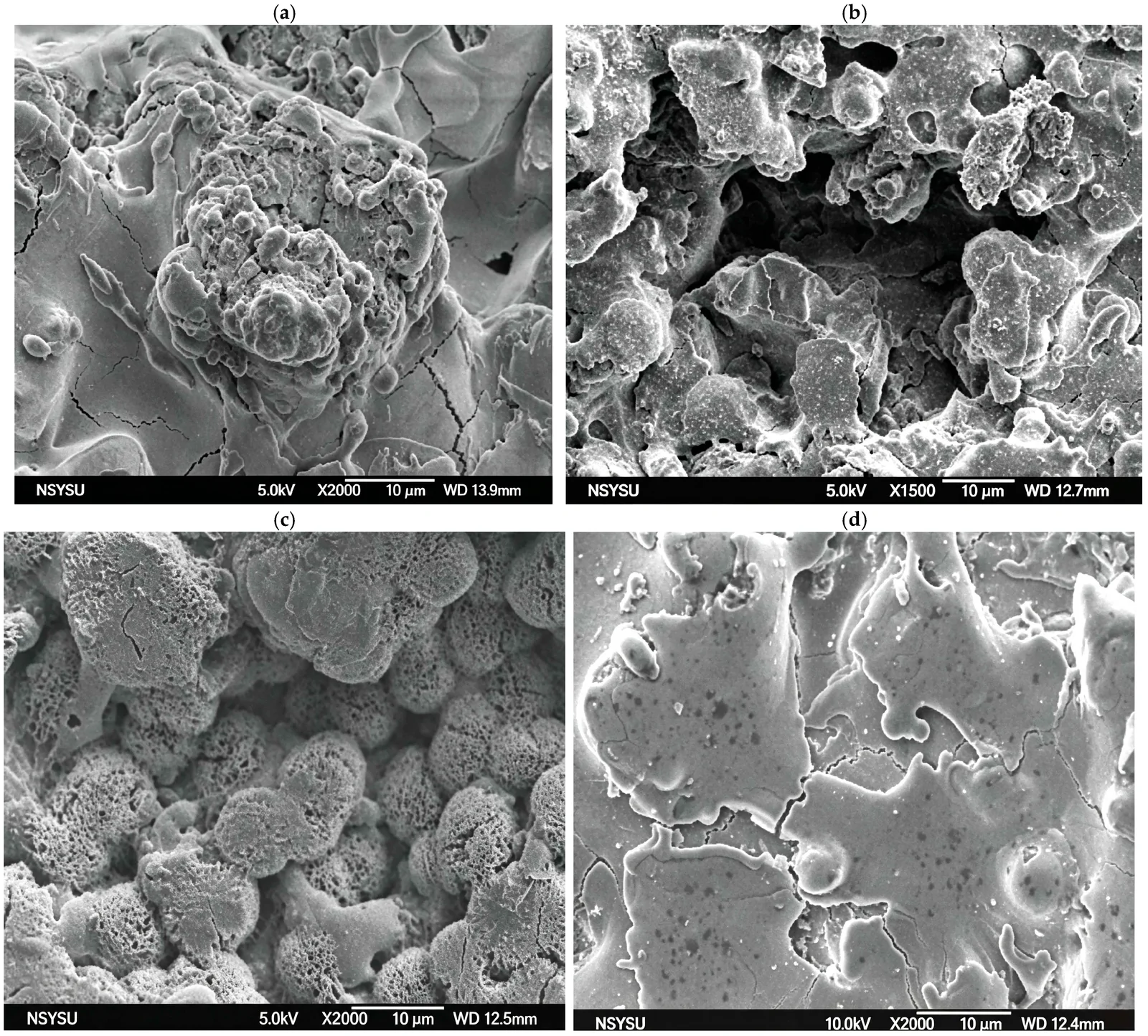
The surface morphology of the ZrO_2_ coating applied to the substrate shown at 1000× magnification for the following tests: (**a**) trial 6, (**b**) trial 9, (**c**) trial 14, and (**d**) trial 16.

As shown in Figure 1, the surface morphology of the ZrO_2_ coating applied to the substrate for tests 6, 9, 14, and 16 in Table 1 is shown at a magnification of approximately 1500–2000. It can be seen that for the coating powder, when the carrier gas flow rate is ionized by the arc formed between the cathode and anode of the APS spray gun, a high-temperature plasma jet is generated; this jet accelerates and melts the injected YSZ powder particles, being sprayed onto the surface in the form of individual particles or droplets, resulting in the formation of partially melted and unmelted fine particles in the zirconia coating. The thickness of the average coating was 84.17 µm, with a standard deviation of 11.18 µm. It is shown in Figure 1a that the microstructure of Test 6 consists of a large, continuous, and rough fused deposit structure, which corresponds to a high-hardness region, whereas numerous radial/reticulated microcracks are exhibited, which is a typical feature of high brittle hardness. This is accompanied by a small number of unmelted or partially melted particles and flower-like crystals, which indicate a region of high wear. In addition, a small number of unmelted or partially melted particles, as well as flower-like crystals, are scattered throughout the bonded area, indicating severe high-speed impact in this region. This is primarily due to stress concentration sources generated during the impact, resulting from differences in thermal expansion coefficients (CTEs), which can easily lead to interlayer delamination and block-like spalling. Consequently, although the microhardness is high, the wear resistance is extremely poor. Figure 1b shows the morphology of test 9, which has a loose coating structure and extremely high porosity. The mean coating thickness was 123 µm, with a standard deviation of 5.48 µm. The splat has not spread enough, which has led to a number of irregular voids and gaps between the layers. The particles are not firmly bonded to each other. The effective bearing area is consequently significantly reduced, leading to extremely low microhardness. Meanwhile, these voids provide initial wear pathways, which result in moderate wear rates. A poor structural density of the coating is caused by too little powder melting and insufficient kinetic energy of particles impacting the surface. Such defects are caused by a mismatch of process conditions during manufacture. There are no advantages evident in its performance compared to other tests. As shown in Figure 1c, test 14 had the lowest microhardness (890 HV) and the highest wear volume losses (12.63 mm^3^) of all the tests in the table. The average coating thickness was found to be 83.32 µm, with a standard deviation of 15.13 µm. Structurally, there is a porous aggregate structure resembling coral or a flower cluster. This consists of spherical or block-shaped aggregates measuring approximately 5–10 μm. These aggregates grow outwards from the centre, forming a daisy-like pattern. A network of microporous channels is visible on the surface of and within the aggregated particles. This structure resembles a sponge or coral, filled with interconnected pores and channels. It exhibits signs of sintering following melting by a high-temperature plasma flame yet still has a large number of partially open pores inside it. This may be due to insufficient heat input, which causes only partial melting or sintering of the internal particles. The formation of numerous pores, coupled with a lack of metallurgical bonding due to insufficient sintering, results in weak internal particle bonding, making the material susceptible to delamination or fracture. Compared to other groups, this results in the most severe defects in this sample. Consequently, the porous structure cannot withstand sustained mechanical loads or wear and tear. The microstructure of test 16 is shown in Figure 1d. This consists of large splats that are fully melted and well-spread. An average coating thickness of 129.33 µm was achieved, with a standard deviation of 6.12 µm. There is a continuous, dense structure with only minor, fine thermal cracks. No unmelted particles or large pores are visible, and the interfaces between the splat layers are tightly bonded. This is attributed to the dense, fused structure, which provides high microhardness. In addition, the coatings exhibit few structural defects and strong adhesion to flat surfaces, which effectively prevents wear and peeling. These results suggest that the appropriate set of spray parameters can achieve complete particle melting, high coating density, and the lowest defect rate, which is key to obtaining high-performance ZrO_2_ coatings that strike a balance between microhardness and wear resistance.

### 4.3. Cross-Sectional Microstructure of Coatings

**Figure 2:**
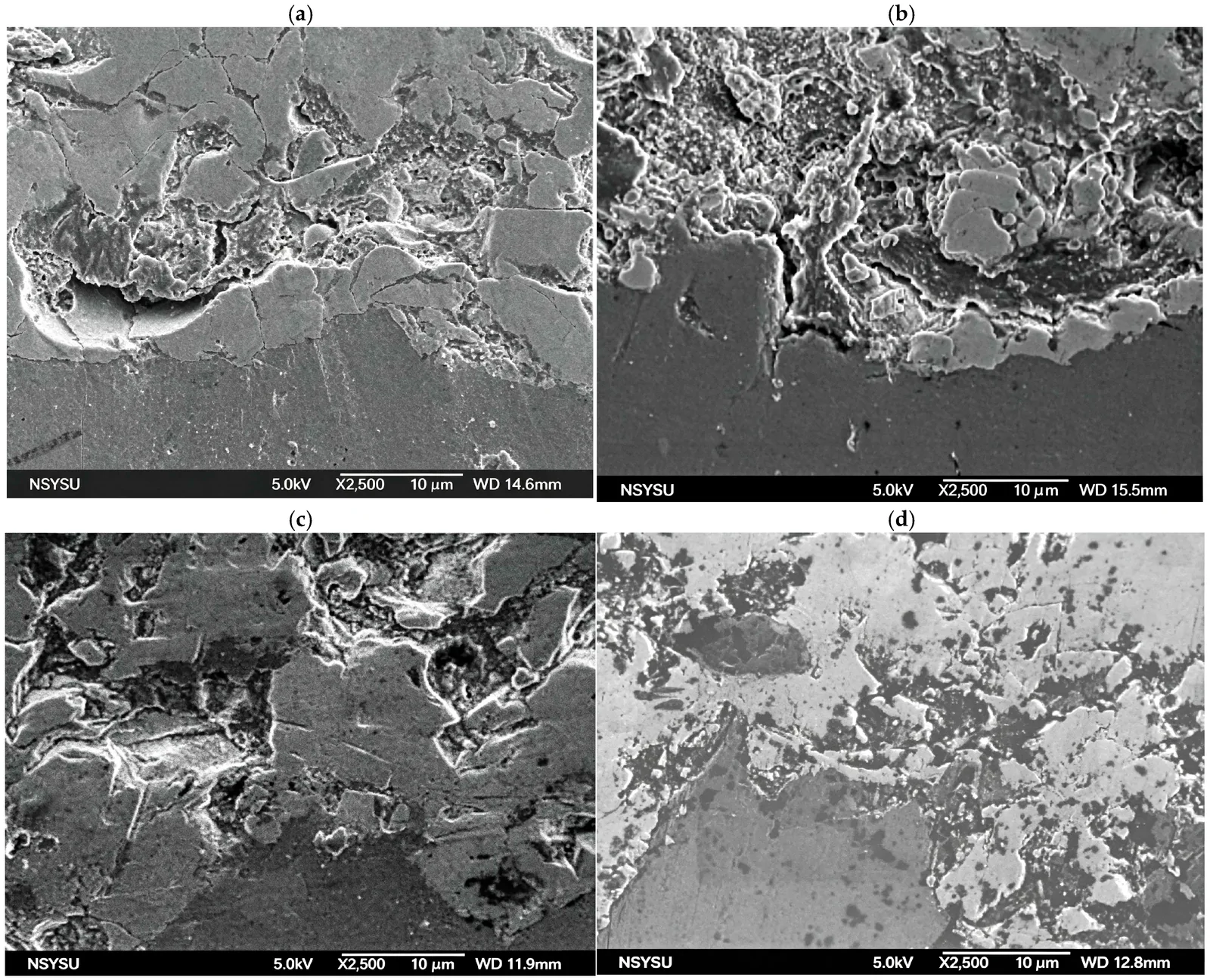
Microstructural distribution in the interface region of plasma-sprayed coatings: (**a**) Trial 6, (**b**) Trial 9, (**c**) Trial 14, and (**d**) Trial 16.

Figure 2 shows the microstructural distribution in the interface region of the plasma-sprayed ZrO_2_ coating. The coating exhibits the typical lamellar structure formed by deformed splats stacking after impacting the substrate. Various defects, such as pores, cracks and unmelted particles, are observed when different spraying parameters are used, and these defects are mainly concentrated near the interface. The microstructural distribution in the interface region is shown in Figure 2a. It reveals discontinuous and intermittent coating interfaces, with distinct voids, unmelted particles, cracks, and gaps. There is no tight mechanical interlocking between the coating and the substrate, and in some areas, debonding has nearly occurred. Such interface structural defects act as stress concentration zones, which degrade the mechanical strength and toughness of the coating. Furthermore, they may lead to rapid cracking and delamination when subjected to thermal shock or external forces. Figure 2b shows that the interfacial region of the coating exhibits a disordered layered structure with weak interlayer bonding, as well as a large number of transverse through-cracks, longitudinal cracks, and network-like microcracks. Numerous unmelted particles, high porosity, and oxide inclusions are concentrated in the interfacial region, resulting in a brittle interlayer that significantly reduces the interfacial bond strength. Figure 2c shows the microstructure of an interface exhibiting moderate defects. The interlocking lines at the interface are continuous but exhibit localized voids and slight delamination, while mechanical interlocking is observed in some areas. The layered stacking structure within the coating is loosely bonded, with microcracks, interlaminar cracks, and short longitudinal cracks visible. As shown in Figure 2d, the microstructure at the interface clearly reveals powder particles embedded in the substrate. The interface between the coating and the aluminium alloy substrate is indistinct, with randomly embedded sprayed particles in the surface layer of the substrate. This interface region appears to form a mechanically interlocking structure. Within the coating, a layered stacking structure is visible, and no interlayer microcracks, short longitudinal cracks, or reticulated microcracks were observed.

### 4.4. Wear Behavior of Coatings

**Figure 3:**
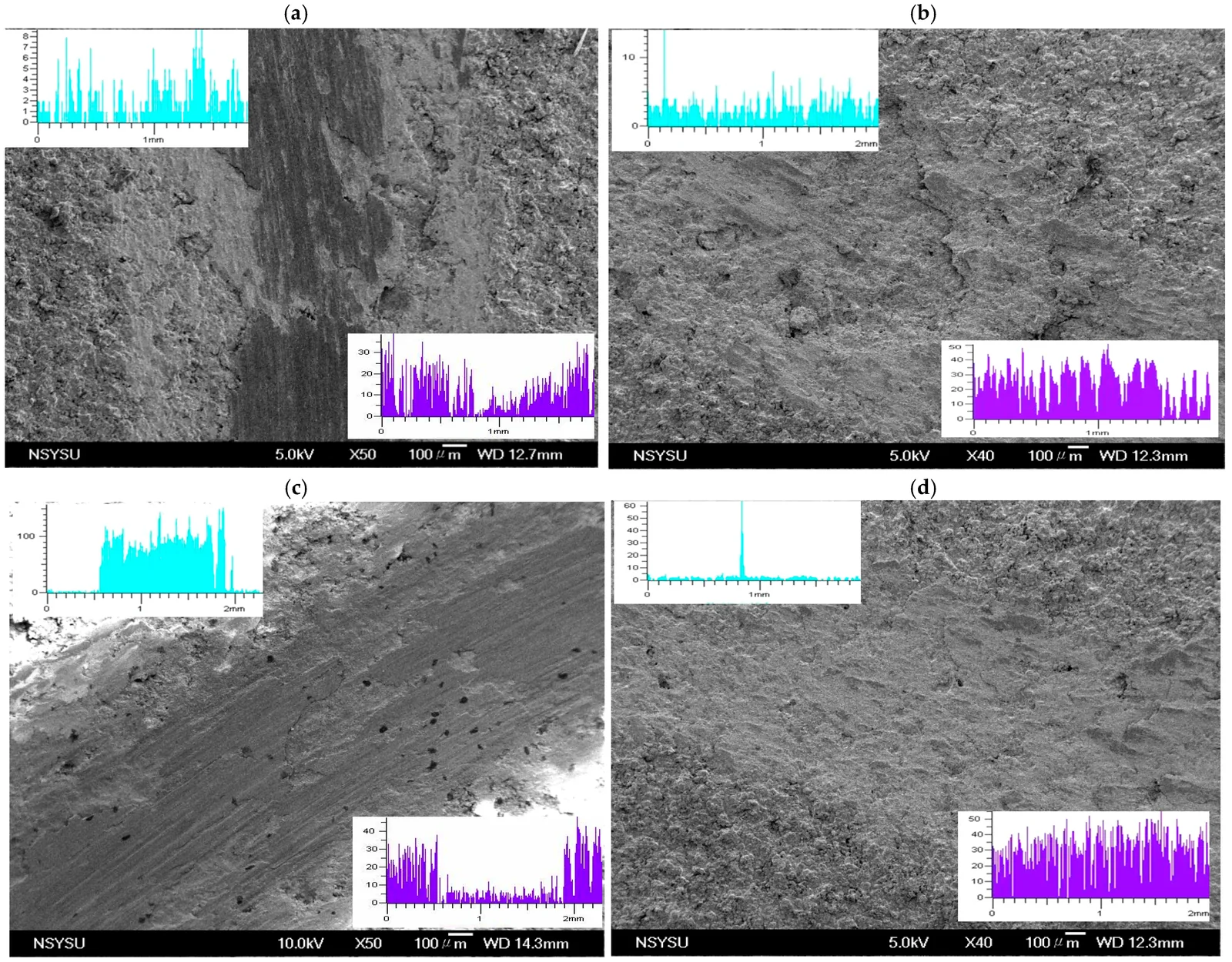
Low-magnification wear morphologies illustrating the influence of different parameters on the wear resistance of coatings: (**a**) Trial 6, (**b**) Trial 11, (**c**) Trial 14, and (**d**) Trial 16. Note that the blue line in the top-left corner shows the distribution of aluminium (Al), while the purple line in the bottom-right corner shows the distribution of zirconium (Zr).

The surface morphology of the plasma-sprayed ZrO_2_ coating subjected to wear testing is shown in Figure 3a. It exhibits visual characteristics of severe spalling wear, with a highly prominent, dark, wide, plastic band-like region in the center. EDS analysis of this region revealed an Al content higher than that of ZrO_2_. This indicates deep friction marks exposed following the separation of the coating from the substrate or internal layers. In addition, the average coefficient of friction of its coating is 0.436 in Test 6, with a volumetric wear rate of 11.19 × 10^−4^ mm^3^/(Nm). It has been established that under these process parameters, extremely poor adhesion is exhibited by the coating. This is due to the fact that the coating is of a soft consistency during the process of abrasion testing. In such cases, microscopic fissures rapidly propagate, leading to the complete delamination of the coating, which is unable to withstand the abrasion load. As Figure 3b illustrates, the overall colour of the wear surface is almost identical to that of the unworn ZrO_2_ structure, with only the ZrO_2_ tracks resulting from wear being visible. It is worth noting that there are no signs of deep wear on the surface; however, pits in the central area are quite noticeable. These pits are caused by particles falling off and becoming loose. The average coefficient of friction of its coating was found to be 0.497 in test 11. This is 3.07 × 10^−4^ mm^3^/(Nm) for the volumetric wear rate. It has been indicated by EDS analysis that the ZrO_2_ content is higher than that of Al, which indicates that the coating appears significantly more compact compared to that shown in Figure 3a. Unlike the unworn coating, Figure 3c shows distinct wear tracks, with the depth of the traces appearing significant and small pinholes in the central area seemingly indicating a loose coating structure. The accumulation of coating material on the substrate surface as a result of wear has caused plastic deformation. The EDS analysis indicates that the Al content is much higher than that of ZrO_2_, which indicates that the coating has been removed. The presence of pores in the unworn coating next to the wear tracks clearly confirms these findings. In addition, a value of 0.439 was found to be the average coefficient of friction of its coating in test 14, which is 12.63 × 10^−4^ mm^3^/(Nm) for volumetric wear rate. This is likely the source of cracks, which are leading to localized delamination. Figure 3d shows the characteristics of uniform wear, with a relatively uniform and fine surface texture. The wear grooves are relatively shallow, and no significant large-scale spalling is observed. Notably, no deep wear marks are visible in this area, nor are the localized pits in the central region as pronounced. Due to its uniform and dense wear pattern, this coating has the lowest wear rate and is therefore the most wear-resistant of the four tests. There was no catastrophic spalling during the wear process. In addition, in test 16, the average coefficient of friction for its coating, which is 3.82 × 10^−4^ mm^3^/(Nm), was found to be 0.453 for volumetric wear rate. This is evidenced by the wear morphology observed in EDS analysis, which shows a high concentration of ZrO_2_ and oxides but no Al. These results indicate that the coating has a dense structure and an interlaced plate-like structure, which further confirms its excellent interfacial and interlayer properties. As can be seen in the above figure, there were no obvious differences between the frictional coefficient curves, but the wear traces exhibited significant differences in pattern. In tests 11 and 16, for example, the track areas had smooth, dense surfaces, demonstrating good tribological properties. By contrast, tests 6 and 14 showed that the track areas had a severely smeared surface and that wear grooves appeared under similar conditions of friction coefficient. Overall, it appears that the coefficient of friction has little effect on tribological properties.

### 4.5. Statistical Variation Estimation Affecting Coating Characteristics

In the study, a statistical analysis was carried out to examine the impacts of different plasma spraying parameters on the microhardness and wear volume losses of ZrO_2_ coatings. This analysis was performed using a statistical method known as analysis of variance (ANOVA). It aimed to identify the key parameters that significantly influence coating performance, while excluding those with negligible effects. These findings will inform subsequent predictions using a response surface modelling approach. Table 2 provides a detailed breakdown of the ANOVA results, including the process parameters (factors), sums of squares, degrees of freedom, variances, F-values and the percentage contribution of each factor for the integrated SNRs. The results of the ANOVA suggest that the four major factors influencing the microhardness and wear volume losses of ZrO_2_ coatings are accelerating voltage (B), stand-off distance (E), powder feed rate (F) and primary gas Ar/H_2_ (H), with a combined contribution of 79.70% (20.63%, 17.50%, 24.97%, and 16.60%, respectively). In contrast, the number of coating layers (A), arc current (C), travel speed (D), and carrier gas flow rate (G) had smaller effects while exhibiting less error (contribution rate < 3%). These results were incorporated into subsequent studies of RSM models.

### 4.6. Construction of the Empirical Model

As shown in Table 1, the configurations of the experimental parameters and levels based on the Taguchi design method, along with the experimental results, are given. The responses of microhardness and volumetric wear loss for plasma-sprayed ZrO_2_ coatings are presented. According to the analysis of variance (ANOVA) in the Taguchi design method, the significant parameters are accelerating voltage (B), stand-off distance (E), powder feeder rate (F), and primary gas Ar/H_2_ (H). These parameters have been included in the regression analysis of the volumetric wear of ZrO_2_ coatings. In this study, the aforesaid parameters were employed for regression modelling, which was constructed using SPSS 26 software based on the data obtained from the experimental design. Each of the different models was used to calculate the results. Models containing linear, interactive and quadratic terms were employed in the RSM. Based on Equation (4), a table of analysis of variance (ANOVA) including linear terms, interaction terms, and quadratic terms was constructed, as shown in Table 2. The four key factors were addressed by us by elucidating their response surfaces for microhardness (Y_1_) and wear volume losses (Y_2_). The coefficient estimates (β) for these models were then calculated by employing regression methods. As a result, the fitted linear, interaction, and quadratic terms are shown in Equations (5)–(7), respectively:

(5) $$Y_{1}=-29295.494-266.131B+1764.181E-59.479F+1156.246-23.335BE-2.264BF+6.296BH+8.866EF+4.324EH-2.962FH+1.496B^{2}-29.814E^{2}+5.644F^{2}-14.150H^{2}$$

Based on Equation (5), the effects of E and H, the BE interaction term, and the H^2^ quadratic term were all significant at the 0.05 level. The effects of the BH interaction term and the F^2^ quadratic term were also significant, but only just (0.05 < *p* < 0.1). The remaining terms had no significant effect (*p* > 0.1). The model was re-fitted by removing all non-significant terms and retaining only the significant and marginally significant terms (E, H, BE, BH, F^2^, H^2^). Backward-stepwise regression was implemented because the full-order quadratic model from the L18 orthogonal array was globally insignificant. The elimination of terms was sequential, with those having a *p*-value higher than 0.10 being removed from the process as shown in Table 3. A reduced quadratic model (Y_1r_) for the microhardness containing significant terms was reconstructed. These terms are B, E, BE and B^2^ and E^2^.

(6) $$Y_{1r}=-38,674.60+83.40B+601.33E+1231.26BE-8.43B^{2}-11.11E^{2}$$

The global significance of the new model was verified by conducting an analysis of variance. Additionally, As shown in Table 4, the BF, BH and EF interaction terms significantly affected wear volume losses (*p* < 0.05), while the main effect of F and the BE interaction term were marginally significant (0.05 < *p* < 0.1). The remaining terms had no significant effect (*p* > 0.1). These results suggest that wear resistance in the coating is primarily governed by the interaction of multiple process parameters. Therefore, optimising the BF, BH and EF interaction terms is the most effective way to improve wear resistance.

(7) $$Y_{2}=-57.857-0.203B+10.314E+5.835F-2.469H-0.154BE-0.083BF+0.076BH+0.241EF-0.116EH-0.050FH$$

Based on Table 3 and Table 4, the experimental data were fitted using the above model. The results of the regression analysis of plasma-sprayed ZrO_2_ coatings showed that the coefficients of determination (R^2^) for the reduced quadratic model of microhardness and the interaction model with wear volume losses were 0.752 and 0.845, respectively. The adjusted R^2^ values were meanwhile found to be 0.649 for microhardness and 0.624 for wear volume losses. The *p*-values for both models were 0.044 and 0.0023 respectively, indicating an excellent fit of these models. Furthermore, the Root Mean Square Error (RMSE) was calculated to be 90.85 HV, which is a small deviation compared to the full hardness test range (890–1510 HV). Also, the RMSE is 1.76 mm^3^, which accounts for only ~14% of the maximum wear volume loss (12.63 mm^3^). These results further confirm the satisfactory accuracy of the fit. Overall, the above equations can be used to predict coating microhardness and wear volume losses for different sets of process parameters. They can be used not only to plot response surface maps that analyse parameter interactions and nonlinear effects but also to optimise process parameters to help ensure that wear volume loss is minimised and microhardness is maximised.

### 4.7. Graphical Analysis of Empirical Models for Wear Volume Properties

**Figure 4:**
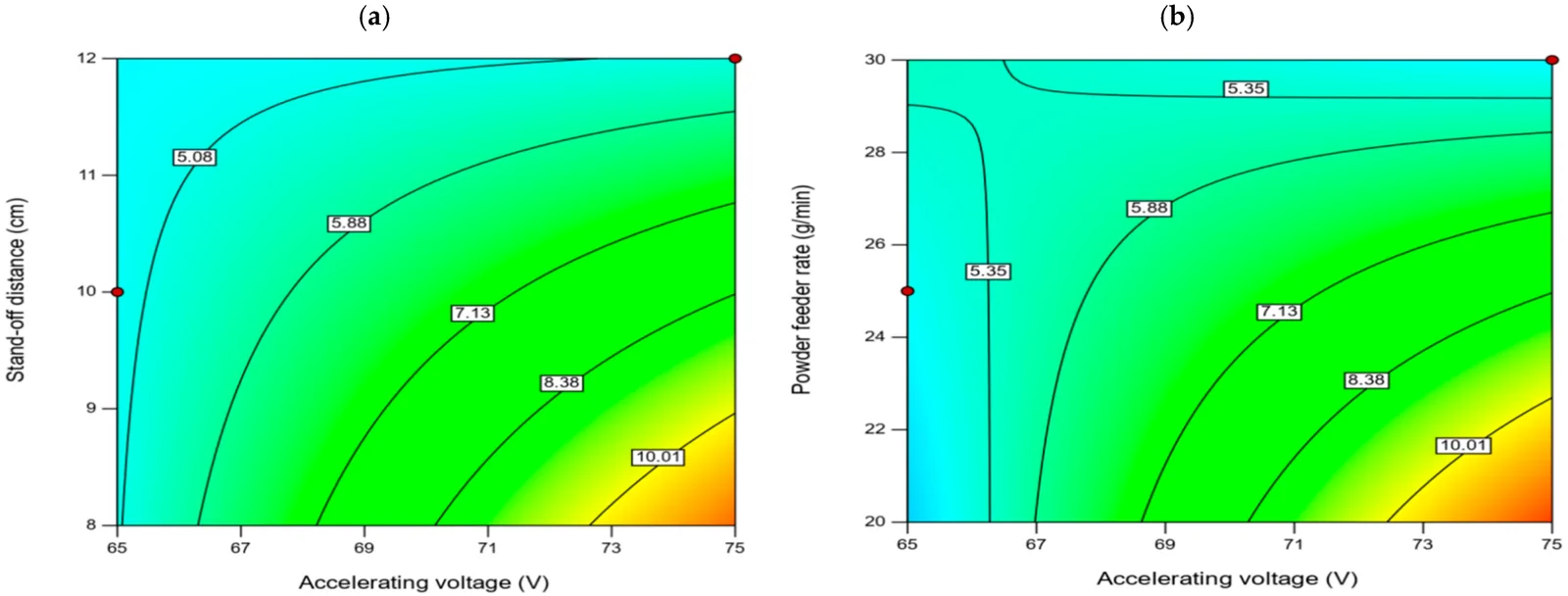

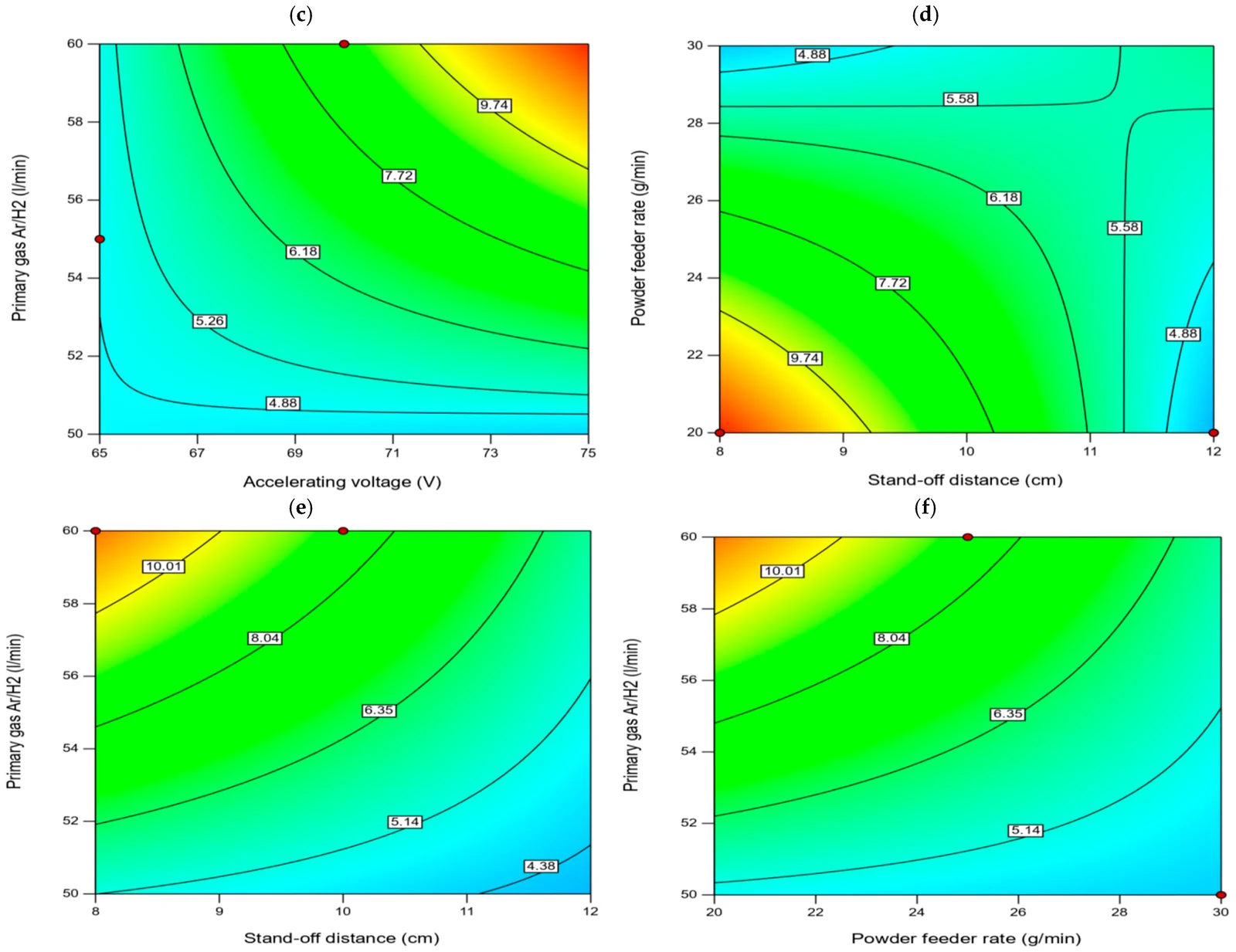
Contour plot of the response surface for wear volume losses yield with the interaction function of the main control factors: (**a**) accelerating voltage and stand-off distance, (**b**) accelerating voltage and powder feeder rate, (**c**) accelerating voltage and primary gas Ar/H_2_, (**d**) stand-off distance and powder feeder rate, (**e**) stand-off distance and primary gas Ar/H_2_, and (**f**) powder feeder rate and primary gas Ar/H_2_. Low, medium and high values are represented by green, blue and red areas, respectively, while the red points represent the boundary vertices of the space for the experimental design.

A more detailed insight into the properties of ZrO_2_ coatings produced by plasma spraying can be obtained by presenting contour plots of the regression functions used in Equations (5)–(7) that relate to wear volume losses and microhardness attributes in the coatings. Figure 4 shows the interaction functions, which depict two-dimensional contour plots of the response surfaces for wear volume loss production, showing how it is influenced by the primary controlling factors. As shown in Figure 4a, a two-dimensional contour plot illustrates the interaction between the accelerating voltage and the stand-off distance on the wear volume losses of the plasma-sprayed coating. The distinct non-parallel distribution of the contour lines confirms the significant interaction between the two parameters (*p* < 0.05). The wear volume loss decreases significantly with a reduction in accelerating voltage and an increase in stand-off distance. The minimum wear volume loss (~5.08 mm^3^) is achieved with a low accelerating voltage (65–67 V) and a high stand-off distance (11–12 cm). Together, these conditions constitute the optimal process window for superior wear resistance. Conversely, combining a high accelerating voltage (73–75 V) with a low stand-off distance (8–9 cm) results in the maximum wear volume losses (~10.01 mm^3^) and should therefore be avoided during process optimisation. These results demonstrate that synergistically regulating the accelerating voltage and stand-off distance is critical to achieving wear-resistant coatings with high durability. The plot in Figure 4b is a two-dimensional contour plot showing a highly significant interaction between the accelerating voltage (B) and the powder feed rate (F) on the wear volume losses of the plasma-sprayed coating, with *p* = 0.016. The diagram illustrates the optimal wear resistance range, which is indicated by the light blue area. This region is characterised by a low accelerating voltage of 65–67 V and a full powder feed rate of 20–30 g/min, and it maintains a low wear volume losses of 5.35–5.88 mm^3^. Conversely, the wear volume losses can also be reduced to 5.35 mm^3^ at high accelerating voltages (73–75 V) and high powder feed rates (28–30 g/min). These two regions represent the optimal parameter windows for the coating’s wear resistance. Figure 4c illustrates a two-dimensional contour plot generated using the response surface method, which shows the interaction between accelerating voltage (B) and primary gas Ar/H_2_ (H) on the wear volume losses of plasma-sprayed coatings. As can be seen from the graph, low accelerating voltage (65–67 V) and low primary gas Ar/H_2_ (50–52 L/min) exhibit the lowest wear volume losses (4.88–5.26 mm^3^), representing the parameter range that yields the best wear resistance for the coating. Under this combination, the coating structure exhibits ideally melted powder, low thermal stress, tight interlayer bonding, and high surface compactness, resulting in optimal wear resistance. The pattern in Figure 4d is similar to that in Figure 4d. It illustrates the interactive effect of stand-off distance (E) and powder feeder rate (F) on the wear volume losses of plasma-sprayed coatings in the form of a 2D contour plot. The powder feed rate increased from 20 g/min to 30 g/min when the spray distance was compared at 8–9 cm and 11–12 cm. The wear volume losses decreased from 9.74 mm^3^ to 4.88 mm^3^, showing a reduction of 49.9%. In contrast, the wear volume losses for the latter remained stable at approximately 4.88 mm^3^, with a fluctuation range of just 14.3%. This suggests that the former demonstrates significantly improved wear resistance compared to the latter when the powder feed rate is increased. The two-dimensional contour plot in Figure 4e illustrates the interaction between the stand-off distance (E) and the primary gas Ar/H_2_ (H) with respect to the wear volume losses of the plasma-sprayed coating. The stand-off distances were 8–9 cm and 11–12 cm. The primary gas Ar/H_2_ was increased from 50 L/min to 60 L/min. The wear volume losses for the former increased from 5.14 mm^3^ to 10.01 mm^3^. This represents an increase of 94.7%. For the latter case, the wear volume losses increased only from 4.38 mm^3^ to 5.14 mm^3^. This represents an increase of just 17.4%. This suggests that when the main gas flow rate increases, the stand-off distance has a more significant impact on wear resistance in the former case. As shown in Figure 4f, the interactive effect of powder feeder rate (F) and primary gas Ar/H_2_ (H) on the wear volume losses of plasma-sprayed coatings is illustrated by a 2D contour graph. The contour lines reveal a clear non-parallel distribution. The optimal wear resistance zone is found in the cyan-blue core area, which corresponds to a high powder feed rate of 28–30 g/min and a low primary gas Ar/H_2_ of 50–52 L/min. This zone has the lowest wear volume losses (5.14 mm^3^), which makes it the parameter window that yields the best wear resistance for the coating. Such conditions result in uniform molten powder, a high-density coating and optimised wear resistance. Based on the 2D contour plot of the response surface, the process parameters that have been found to minimise the wear volume losses of the plasma-sprayed coating are as follows: an accelerating voltage of 65–67 V; a stand-off distance of 11–12 cm; a primary gas Ar/H_2_ of 50–52 L/min; and a powder feed rate of 28–30 g/min. The corresponding predicted minimum wear volume loss is 4.88–5.08 mm^3^. This result was confirmed by a regression model, with a calculation error of less than 2%.

### 4.8. Graphical Analysis of Empirical Models for Hardness Properties

**Figure 5:**
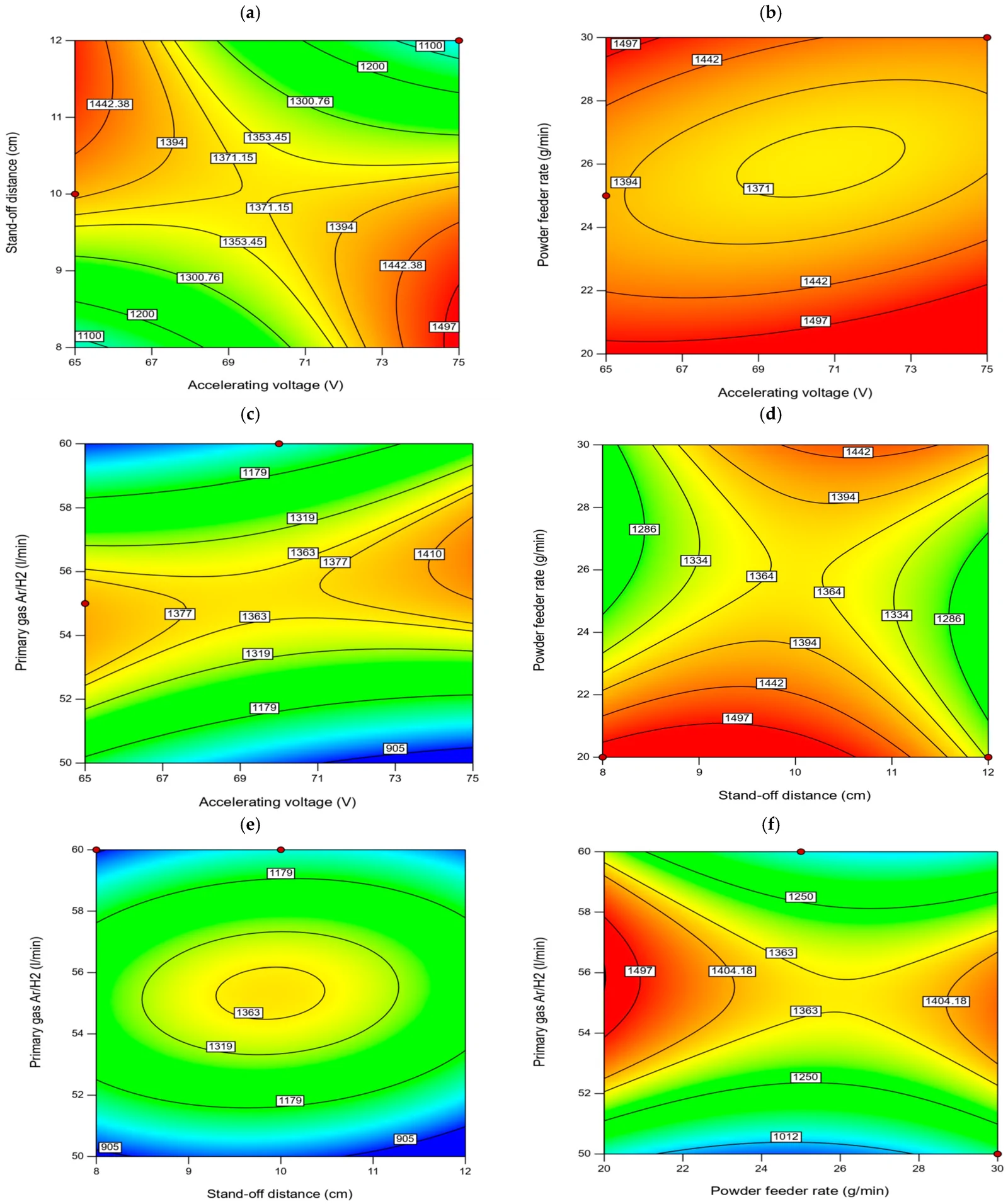
Contour plot of the response surface for microhardness yield with the quadratic function of the main control factors: (**a**) accelerating voltage and stand-off distance, (**b**) accelerating voltage and powder feeder rate, (**c**) accelerating voltage and primary gas Ar/H_2_, (**d**) stand-off distance and powder feeder rate, (**e**) stand-off distance and primary gas Ar/H_2_, and (**f**) powder feeder rate and primary gas Ar/H_2_. The representation of low, medium and high values is by the green, blue and red areas, respectively, while the red points represent the boundary vertices of the space for the experimental design.

Figure 5 presents a two-dimensional contour plot of the quadratic response surface function for the microhardness of the coating as a function of the four main control variables in plasma spraying. It visually illustrates the relationship between the interactions of these four key process parameters and microhardness, where higher microhardness values indicate superior mechanical properties of the coating. As shown in Figure 5a, the two-dimensional contour plot displays the effects of accelerating voltage and spray distance on the microhardness of the plasma-sprayed coating. The microhardness increases significantly as the accelerating voltage rises and the spraying distance decreases. The optimal parameter zone lies between an accelerating voltage of 73–75 V and a spraying distance of 8–9 cm, where the highest microhardness reaches 1497 HV. Conversely, the lowest microhardness zone occurs at an accelerating voltage of 65 V and a spraying distance of 8–12 cm, where the minimum microhardness is only 1100 HV. The 2D contour plot in Figure 5b illustrates the significant interactive effect of accelerating voltage (B) and powder feeder rate (F) on the microhardness of plasma-sprayed coatings. The findings demonstrate that the most minimal microhardness (roughly 1371 HV) was achieved at a powder feed speed of 24–26 g/min and an accelerating voltage of 69–71 V. Conversely, the highest microhardness (~1497 HV) was produced by the combination of a low powder feed rate (20–22 g/min) and the full range of accelerating voltages (65–75 V). These conditions were identified as the optimal process parameters for achieving superior mechanical properties. The results suggest that adjusting the accelerating voltage and powder feed rate appropriately is essential for producing high-performance coatings with high microhardness. The contour plot in Figure 5c shows a saddle curve of coating microhardness, where the independent variables are accelerating voltage (B) and primary gas Ar/H_2_ (H). The results show that the highest microhardness (approx. 1410 HV) is achieved when using a combination of high accelerating voltage (73–75 V) and high primary gas Ar/H_2_ (58–60 L/min), which is attributed to sufficient powder melting and high particle impact intensity. Conversely, the lowest microhardness (approx. 905 HV) was observed with low accelerating voltage (65 V) and low primary gas Ar/H_2_ (50–52 L/min), which was attributed to insufficient melting and high porosity. The contour plots exhibited a typical saddle-shaped pattern, further confirming the strong interaction between these two process parameters. As shown in Figure 5d, a saddle curve is represented by a two-dimensional contour plot, which illustrates the interaction between stand-off distance (E) and powder feed rate (F) with regard to the microhardness of the coating. The patterns shown in Figure 5a,c were approximated, wherein the contour curves exhibited a saddle-shaped profile, confirming the strong interactive effect between the two parameters. An increase in stand-off distance from 8 cm to 12 cm, when the feed rate was 25 g/min, resulted in a 14.1% increase in microhardness. The highest microhardness (approx. 1497 HV) was observed at a stand-off distance of 8–9 cm and a powder feed rate of 20–22 g/min. In contrast, the combination of a stand-off distance of 11–12 cm and a powder feed rate of 28–30 g/min resulted in lower microhardness (approx. 1286 HV). These results suggest that adjusting the stand-off distance and powder feed rate appropriately is crucial for achieving high microhardness in the coatings. As shown in Figure 5e, the relationship between the stand-off distance (E) and the primary gas Ar/H_2_ (H) with respect to microhardness is illustrated by the contours of the elliptical shape. The contour lines form a typical elliptical pattern. There is a distinct peak in microhardness at the centre. The highest microhardness (approximately 1363 HV) was achieved using the optimal combination of a stand-off distance of 10 cm and a primary gas Ar/H_2_ of 56–58 L/min. This is due to the high gas flow rate ensuring sufficient powder melting and intense particle impact on the surface. Conversely, the lowest microhardness (approx. 905 HV) occurred at distances of 8 cm or 11–12 cm, coupled with low gas flow rates of 50–52 L/min. This is due to insufficient powder melting and low kinetic energy upon impact with the surface, resulting in poor powder melting, high porosity and weak bonding. The contour plot of microhardness, as affected by powder feeder rate and primary gas Ar/H_2_, is shown in Figure 5f. There is a significant increase in microhardness with an increase in primary gas Ar/H_2_ and a decrease in powder feeder rate. At a low powder feed rate (20 g/min) combined with a high main gas flow rate (60 L/min), the highest microhardness (~1497 HV) can be achieved. These results indicate a dense coating structure, which is likely due to thorough powder melting, interface interlocking, and minimal surface defects. Overall, the optimal process parameters to maximize the microhardness of the plasma-sprayed coating are determined based on the two-dimensional contour plot of the response surface. These are as follows: an accelerating voltage of 73–75 V, a stand-off distance of 8–9 cm, a primary gas Ar/H_2_ of 58–60 L/min, and a powder feed rate of 20–22 g/min. This suggests that the physical mechanism underlying the coating structure is that high voltage increases the energy of the flame, allowing the powder to melt completely. Meanwhile, the close stand-off distance increases the velocity of the particles impacting the surface, thereby enhancing the structural densities and interface interlocking properties of the coating. This significantly improves the microhardness of the coating.

### 4.9. Confirmation Experiments

**Figure 6:**
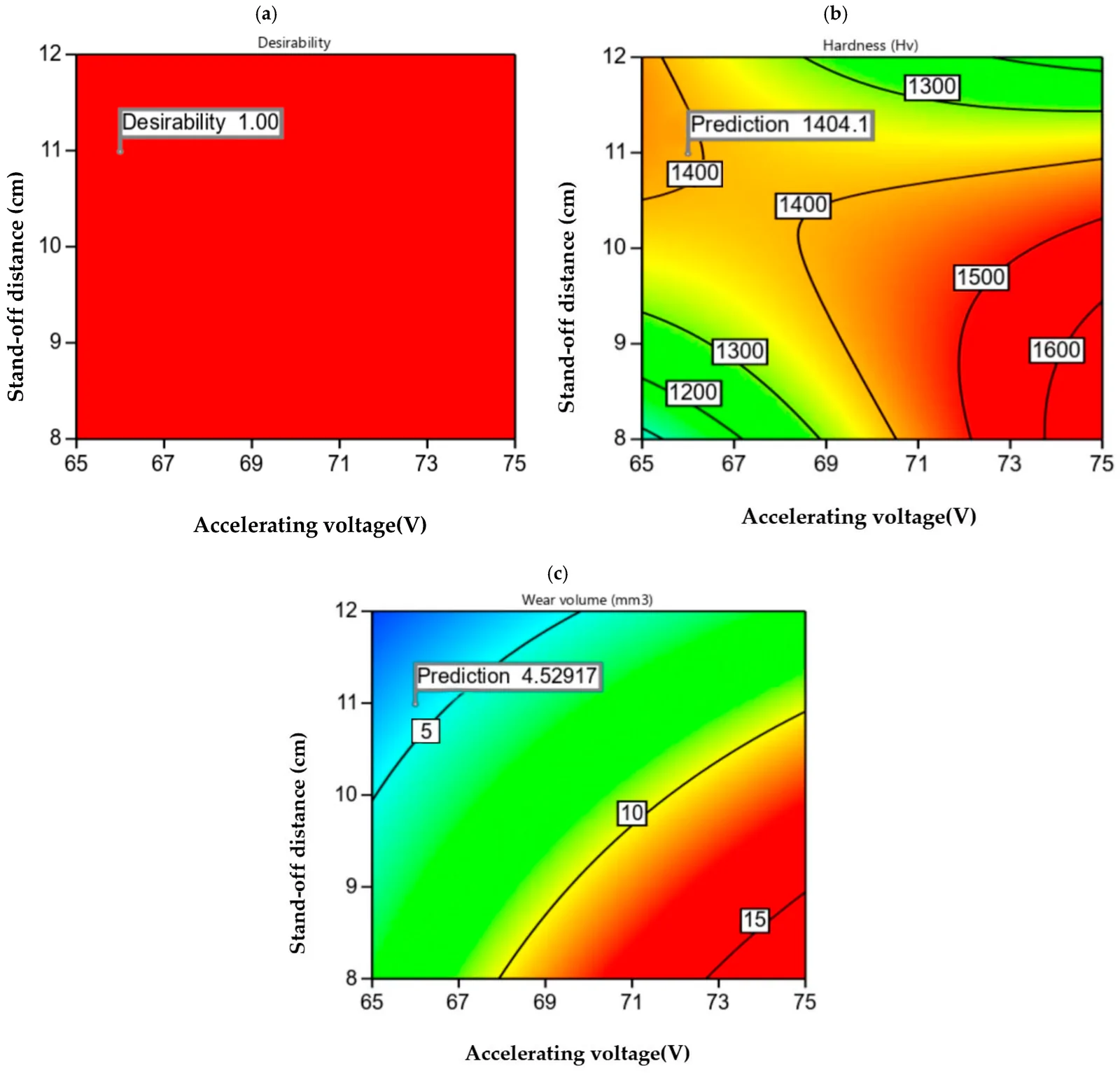
The contour plot shows (**a**) the desirability, (**b**) the microhardness, and (**c**) the wear volume of the plasma-sprayed ceramic coating. The representation of low, medium and high values is by the green, blue and red areas, respectively.

**Figure 7:**
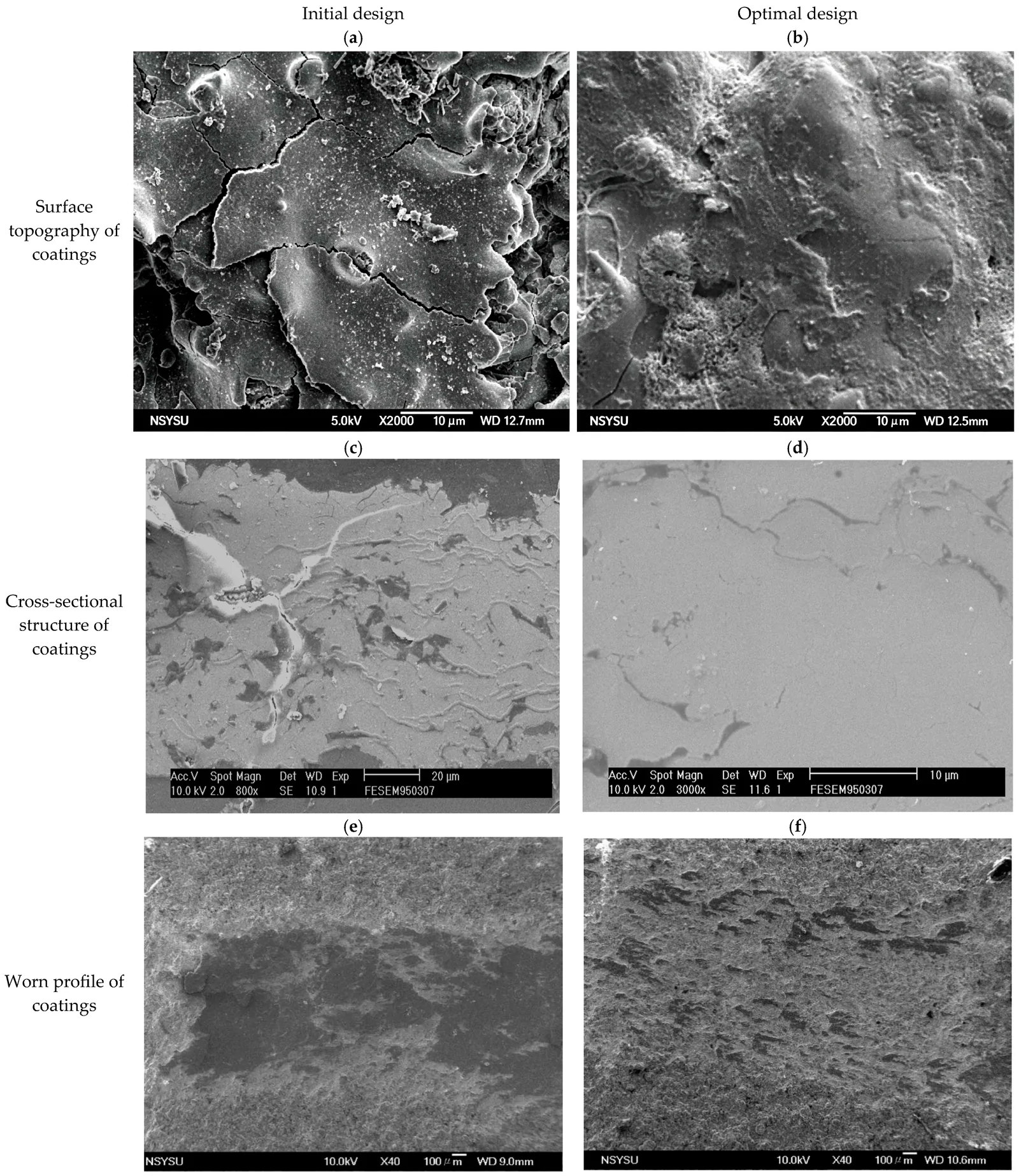
SEM images of cross-sectional, surface topography and worn structures of plasma-sprayed ZrO_2_ coatings: (**a**,**c**,**e**) initial test and (**b**,**d**,**f**) optimal test for multiple characteristics.

To more effectively validate the statistical model and gain a better understanding of the properties of microhardness and wear in coatings, we employed a variance-based method to identify a set of optimized parameters, namely A_2_B_1_C_1_D_1_E_2_F_3_G_3_H_2_. The key parameters (B, E, F, H) are shown in Figure 6, which illustrates the curves of microhardness versus wear volume losses as a desirability approaches its maximum value of 1. A desirability score falls between 0 and 1. A score closer to 1 indicates better performance, but the influence of coating parameters on individual properties and multi-response characteristics varies significantly. These individual results, however, do not allow for the determination of the mechanical properties of the coating, nor are they sufficient to meet industrial requirements. By constructing a regression model and combining it with contour and response surface plots, the relationship between processing parameters and coating performance was revealed, which resolved the conflicting requirements inherent in multiple attributes of quality. Furthermore, the desirability-overlapped method was employed to identify the optimal parameter window that satisfies both high hardness requirements and excellent wear resistance. As shown in Figure 6, the highest microhardness was achieved in the range of 73–75 V and 8–9 cm. Conversely, the lowest wear volume losses was observed in the range of 65–67 V and 11–12 cm. However, the influence of coating parameters on individual properties and multifunctional characteristics varies significantly. Therefore, a desirability approach based on the RSM was adopted. As shown in Figure 6a, the contour plot illustrates the desirability function for the plasma-sprayed ceramic coating, which resolves the incompatibility between achieving maximum microhardness and minimum wear volume losses. Based on the contour plots and the results of multi-objective optimization, the optimized process parameters for the plasma-sprayed ceramic coating are as follows: 66 V accelerating voltage, 11 cm stand-off distance, 22 g/min powder feed rate, and 57 L/min primary gas Ar/H_2_. Under these conditions, the predicted microhardness and wear volume loss properties for the contour plots are 1404.1 Hv (Figure 6b) and 4.529 mm^3^ (Figure 6c), respectively, with a desirability of 1.00, indicating a well-balanced solution between high microhardness and excellent wear resistance. Furthermore, a wide range of optimized process parameters (65–75 V, 8–12 cm) at a powder feed rate of 22 g/min and a primary gas Ar/H_2_ of 57 L/min yields numerous parameter combinations that satisfy a desirability of 1.00, which is beneficial for industrial mass production. Confirmatory experiments were conducted under the predicted optimal conditions to verify the accuracy and reliability of the optimization results. By conducting experiments that included microstructural characterization, phase analysis, microhardness measurements, and wear tests, we verified the accuracy and validity of the established model. The results of the prediction were validated by experiments under the optimal conditions described above. The microhardness value obtained after five replicate measurements was 1378.7 ± 65 HV, which compared favourably with the predicted value of 1404.1 HV, a value that represented a percentage error of just 1.84%. Moreover, the value obtained after three values of the wear volume losses was 4.682 mm^3^, while the predicted value was 4.529 mm^3^. This represents a percentage error of 3.27%. Furthermore, the predicted hardness value is 1404.1 HV and the 95% prediction interval lies between 1362.7 and 1445.5 HV. In addition, the predicted value of the wear volume loss is 4.53 mm^3^, with a 95% prediction interval of 4.31–4.75 mm^3^. The narrow 95% prediction intervals show that the uncertainty of the model is low. The above validation results demonstrate a high degree of agreement, as shown by the fact that the results are very similar and there is a clear pattern to be seen. Furthermore, the SNRs for microhardness and wear volume losses in Figure 6 are similar to those in trial 16, which performed well in the orthogonal array. This indicates that several properties of the plasma-sprayed ZrO_2_ coating have been significantly improved. Notably, as can be seen in Figure 7b,d,f, the microstructure of the optimal coating appears to be fully fused and fine-grained, with minimal porosity and cracking. Only minor pitting and small flaking were observed in the worn areas, with a significant portion of the original sprayed structure retained. This results in an intact overall wear surface compared to the initial test (Figure 7a,c,e). This study concludes that the optimised coating prepared using synthetic ZrO_2_ powder exhibits superior surface morphological and structural characteristics of coatings, as observed by SEM. As mentioned above, optimising controlled factors enables the production of high-quality coatings and demonstrates sufficient robustness against noise effects, thereby improving reproducibility.

## 5. Conclusions

This study has been successfully carried out by integrating the RSM with the desirability-overlapped method, which addresses the multi-objective optimization of atmospheric plasma spraying parameters for zirconia coatings, while simultaneously enhancing microhardness and reducing wear loss. Based on the experimental results, the following conclusions can be drawn:

1. An ANOVA analysis was applied to a Taguchi L18 orthogonal array to quantify experimental variation and identify the four dominant spraying parameters. These were spray power, spray distance, the powder feed rate and the carrier gas flow rate. Together, these parameters contribute nearly 79.70% of the total performance variance.
2. The four key parameters in regression models were investigated in terms of their individual effects and interactive influences on the microstructure, mechanical and tribological properties of ZrO_2_ coatings.
3. The regression models demonstrate excellent model fit, as evidenced by the high determination coefficients: 0.845 (*p* = 0.044) for the reduced model of the wear volume losses and 0.752 (*p* = 0.0023) for the quadratic model of the microhardness. Together, these findings underscore the reliability of the model predictions.
4. The well-fitted models are used to plot response surface diagrams for analysing factor interaction and nonlinear effects and to further conduct multi-objective parameter optimisation to simultaneously maximise microhardness and minimise wear volume losses.
5. SEM examination shows that the coating produced using the optimised parameters has a dense microstructure with few pores and cracks, displaying a typical layered structure and uniform surface morphology. The significant enhancement in microhardness and wear resistance can be attributed to the precise model predictions and the optimised spraying parameters.
6. The results confirm that the integrated multi-objective optimisation strategy effectively improves the performance of plasma-sprayed ceramic coatings, providing durable surface protection for industrial components under severe wear conditions. This study also demonstrates that RSM is an efficient and reliable method for optimising the properties of thermal spray coatings.

## Figures and Tables

**Table 1:** The results of experimental tests for an orthogonal array based on Taguchi designs, including the number of control factors, their properties and performance measures. (A) Number of coating layers: 6 and 8; (B) Accelerating voltage (V): 65, 70, and 75; (C) Arc current (A): 550, 600, and 650; (D) Travel speed (mm/s): 300, 400, and 500; (E) Stand-off distance (cm): 8, 10, and 12; (F) Powder feeding rate (g/min): 20, 25, and 30; (G) Carrier gas flow rate (L/min): 20, 25, and 30; (H) primary gas Ar/H_2_ (L/min): 50, 55, and 60.

No. of Tests	Control Factors								Performance Estimate						
	A	B	C	D	E	F	G	H	Microhardness (HV)			Wear Volume Losses (mm^3^)			Integrated
									Mean	St. Dev	SNR	Mean	St. Dev	SNR	SNR
1	5	65	550	20	8	20	5	50	1016	231	59.66	4.79	0.335	−13.61	46.05
2	5	65	600	25	10	25	6	55	1396	248	62.08	6.72	0.354	−16.56	45.52
3	5	65	650	30	12	30	7	60	1223	241	62.23	5.26	0.393	−14.44	47.79
4	5	70	550	20	10	25	7	60	1122	131	61.58	8.39	0.140	−18.48	43.10
5	5	70	600	25	12	30	5	50	1108	137	61.43	8.09	0.180	−18.16	43.27
6	5	70	650	30	8	20	6	55	1510	513	61.94	11.19	0.207	−20.98	40.96
7	5	75	550	25	8	30	6	60	1176	154	62.31	9.28	0.376	−19.35	42.96
8	5	75	600	30	10	20	7	50	1014	288	59.96	5.47	0.472	−14.78	45.18
9	5	75	650	20	12	25	5	55	958	151	59.77	5.63	0.124	−15.01	44.76
10	8	65	550	30	12	25	6	50	1216	317	61.80	5.00	0.270	−13.99	47.81
11	8	65	600	20	8	30	7	55	1151	159	61.39	3.07	0.761	−9.91	51.48
12	8	65	650	25	10	20	5	60	1133	136	61.55	4.91	0.163	−13.82	47.73
13	8	70	550	25	12	20	7	55	1346	287	62.37	5.17	0.116	−14.28	48.09
14	8	70	600	30	8	25	5	60	890	162	59.59	12.63	0.888	−22.04	37.55
15	8	70	650	20	10	30	6	50	1016	76	60.69	3.74	0.482	−11.51	49.18
16	8	75	550	30	10	30	5	55	1490	98	64.18	3.82	0.301	−11.65	52.53
17	8	75	600	20	12	20	6	60	1174	247	61.77	10.29	0.409	−20.25	41.52
18	8	75	650	25	8	25	7	50	1035	184	59.69	8.59	0.428	−18.68	41.01

**Table 2:** A detailed analysis of the analysis of variance, including process parameters, sums of squares, degrees of freedom, mean square, F-values, and the percentage contribution of each factor for the Integrated SNRs.

Control Factors	Sum of Squares	Degrees of Freedom	Mean Square	F-Value	Percent Contribution
A	16.646	1.0	16.646	10.211	6.44
B	53.341	2.0	26.671	16.360	20.63
C	21.521	2.0	10.761	6.601	8.32
D	4.729	2.0	2.365	1.451	1.83
E	45.259	2.0	22.630	13.881	17.50
F	64.571	2.0	32.286	19.804	24.97
G	6.326	2.0	3.163	1.940	2.45
H	42.931	2.0	21.466	13.167	16.60
Error	3.260	2.0	1.630	1.000	1.26
Total	258.587	17.0	15.211		100.00

**Table 3:** The results of the statistical analysis for the reduced quadratic model of microhardness, including the regression coefficient significance table and ANOVA table for the microhardness of the coatings.

**Term**	**Coefficient Estimate**	**Degree of Freedom**	**Standard Error**	**t-Stat**	**Prob > F**
Intercept	−38,674.60	-	7077.56	−5.46	0.00
B	83.40	1	34.74	2.40	0.03
E	601.33	1	240.72	2.50	0.03
BE	1231.26	1	213.73	5.76	0.00
B^2^	−8.43	1	3.43	−2.46	0.03
E^2^	−11.11	1	1.94	−5.72	0.00
**Source**	**Degree of Freedom**	**Sum of Squares**	**Mean Square**	**F-Value**	**Prob > F**
Regression	5	301,241.2	60,248.24	7.299594	0.002355
Residual	12	99,043.72	8253.644		
Cor Total	17	400,284.9			

**Table 4:** The results of the statistical analysis for the interaction model of wear volume losses, including the regression coefficient significance table and ANOVA table for the wear volume losses of the coatings.

**Term**	**Coefficient Estimate**	**Degree of Freedom**	**Standard Error**	**t-Stat**	**Prob > F**
Intercept	0.058	-	128.394	−0.451	0.666
B	−57.857	1	1.533	−0.132	0.898
E	−0.203	1	5.896	1.749	0.124
F	10.314	1	2.711	2.152	0.068
H	5.835	1	2.317	−1.066	0.322
BE	−2.469	1	0.077	−2.014	0.084
BF	−0.154	1	0.030	−2.776	0.027
BH	−0.083	1	0.030	2.511	0.040
EF	0.076	1	0.076	3.174	0.016
EH	0.241	1	0.074	−1.574	0.159
FH	−0.116	1	0.029	−1.748	0.124
**Source**	**Degree of Freedom**	**Sum of Squares**	**Mean Square**	**F-Value**	**Prob > F**
Regression	10	119.344	11.934	3.823	0.044
Residual	7	21.853	3.122		
Cor Total	17	141.197			

## Data Availability

The original contributions presented in this study are included in the article. Further inquiries can be directed to the corresponding authors.
